# Superior Properties of N-Acetylcysteine Ethyl Ester over N-Acetyl Cysteine to Prevent Retinal Pigment Epithelial Cells Oxidative Damage

**DOI:** 10.3390/ijms22020600

**Published:** 2021-01-09

**Authors:** Gian Marco Tosi, Daniela Giustarini, Lorenzo Franci, Alberto Minetti, Francesco Imperatore, Elena Caldi, Paolo Fiorenzani, Anna Maria Aloisi, Anna Sparatore, Ranieri Rossi, Mario Chiariello, Maurizio Orlandini, Federico Galvagni

**Affiliations:** 1 Ophthalmology Unit of the Department of Medicine, Surgery and Neuroscience, University of Siena, 53100 Siena, Italy; gianmarco.tosi@unisi.it; 2 Department of Biotechnology, Chemistry and Pharmacy, University of Siena, 53100 Siena, Italy; daniela.giustarini@unisi.it (D.G.); alberto.minetti@student.unisi.it (A.M.); elenac84@hotmail.it (E.C.); ranieri.rossi@unisi.it (R.R.); 3 Core Research Laboratory, Istituto per lo Studio, la Prevenzione e la Rete Oncologica (ISPRO), 53100 Siena, Italy; lorenzofranci4@gmail.com (L.F.); imperatore@ifc.cnr.it (F.I.); mario.chiariello@cnr.it (M.C.); 4 Istituto di Fisiologia Clinica, Consiglio Nazionale delle Ricerche, 53100 Siena, Italy; 5 Department of Medicine, Surgery and Neuroscience, University of Siena, 53100 Siena, Italy; paolo.fiorenzani@unisi.it (P.F.); annamaria.aloisi@unisi.it (A.M.A.); 6 Department of Pharmaceutical Sciences, University of Milan, 20133 Milan, Italy; anna.sparatore@unimi.it

**Keywords:** oxidative stress, retinopathy, retinal pigment epithelium, age-related macular degeneration, N-acetyl-L-cysteine, N-acetyl-L-cysteine ethyl ester

## Abstract

Oxidative stress plays a key role in the pathophysiology of retinal diseases, including age-related macular degeneration (AMD) and diabetic retinopathy, which are the major causes of irreversible blindness in developed countries. An excess of reactive oxygen species (ROS) can directly cause functional and morphological impairments in retinal pigment epithelium (RPE), endothelial cells, and retinal ganglion cells. Antioxidants may represent a preventive/therapeutic strategy and reduce the risk of progression of AMD. Among antioxidants, N-acetyl-L-cysteine (NAC) is widely studied and has been proposed to have therapeutic benefit in treating AMD by mitigating oxidative damage in RPE. Here, we demonstrate that N-acetyl-L-cysteine ethyl ester (NACET), a lipophilic cell-permeable cysteine derivative, increases the viability in oxidative stressed RPE cells more efficiently than NAC by reacting directly and more rapidly with oxidizing agents, and that NACET, but not NAC, pretreatment predisposes RPE cells to oxidative stress resistance and increases the intracellular reduced glutathione (GSH) pool available to act as natural antioxidant defense. Moreover, we demonstrate the ability of NACET to increase GSH levels in rats’ eyes after oral administration. In conclusion, even if experiments in AMD animal models are still needed, our data suggest that NACET may play an important role in preventing and treating retinal diseases associated with oxidative stress, and may represent a valid and more efficient alternative to NAC in therapeutic protocols in which NAC has already shown promising results.

## 1. Introduction

Age-related macular degeneration (AMD) is considered one of the main causes of severe vision loss among the elderly in the United States and Western societies. The dry form of AMD, also known as atrophic AMD, is characterized by the death of the retinal pigment epithelium (RPE) cells and photoreceptors at the macular level. RPE cells are highly specialized, polarized epithelial cells whose apical side is in intimate contact with the outer segments of photoreceptors. 

Oxidative stress-related damage to RPE cells and photoreceptors is considered an early event in the pathogenesis of AMD [1]. The RPE is essential for retinal function and the integrity of photoreceptors through phagocytosis of the shed tips of the outer segments of photoreceptors, containing the waste products from photo-oxidative damage, such as lipofuscin [2]. Lipofuscin is an undegradable lipid–protein aggregate that accumulates in RPE cells as an organism ages. In RPE cells, the major component of lipofuscin is the pyridinium bisretinoid A2E, a photo-inducible reactive oxygen species (ROS) generator [3,4]. A2E intracellular accumulation enhances RPE photosensitization, generating high levels of both hydrogen peroxide (H_2_O_2_) and superoxide anion (O_2_^• −^) when the cells are exposed to blue-violet light, providing a possible cellular mechanism to explain the RPE dysfunction that causes AMD [5]. In addition, physiological oxidative stress [6] and environmental inducers, such as cigarette smoking and intense sunlight exposure [7,8,9], enhance ROS generation and are related to the development of AMD. Based on these observations, it has been proposed that antioxidants may prevent cellular damage in the retina by reacting with ROS, and dietary supplementation of antioxidant vitamins and minerals may reduce the risk of progression of AMD. The efficacy of such treatments remains controversial and genetic background may play a significant role [10,11]. Therefore, there is a clear need to investigate additional and more effective antioxidants to treat or prevent AMD progression. Among antioxidants, N-acetyl-L-cysteine (NAC) is widely studied and has recently been proposed to have therapeutic benefit in treating AMD by mitigating oxidative damage in the RPE [12]. However, NAC is a low lipophilic compound, so it has poor cell permeability. We previously demonstrated that the N-acetyl-L-cysteine ethyl ester (NACET) possesses more favorable pharmacokinetic properties than NAC itself, both in terms of cellular permeability and bioavailability in vivo, where it is able to cross the blood–brain barrier [13], and as a glutathione (GSH) enhancer in human primary endothelial cells [14]. In fact, NACET rapidly enters the cells where it is immediately de-esterified to NAC, which is subsequently slowly de-acetylated, providing the cells with a continuous source of cysteine, the precursor of GSH, a major component of the antioxidant defense system of mammalian cells. Therefore, we hypothesized that NACET would be an extremely effective antioxidant treatment and a better protective agent than NAC in AMD. To test this hypothesis, in this study, we evaluated NACET’s antioxidant potential in comparison to NAC in ARPE-19 RPE cells, and its capacity to induce GSH enhancement in RPE cells in vitro and in rats’ eyes following oral administration.

## 2. Results

### 2.1. NACET Protects RPE Cells from Oxidative Stress More Efficiently Than NAC

**Figure 1:**
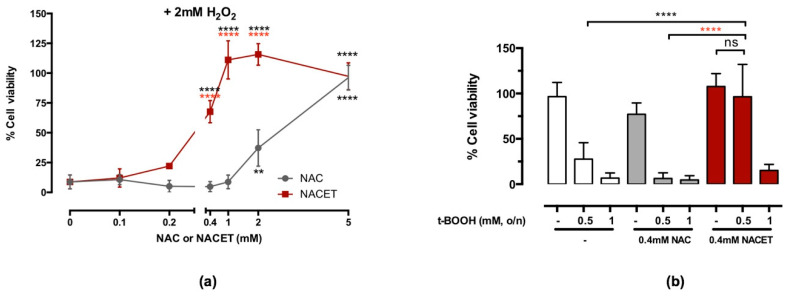
N-acetyl-L-cysteine ethyl ester (NACET) protects retinal pigment epithelium (RPE) cells from oxidative stress. Confluent ARPE-19 RPE cells were treated with increasing concentrations of N-acetyl-L-cysteine (NAC) or NACET for 24 h before viability was measured. Then, 2 mM H_2_O_2_ (**a**) or 0.5 or 1 mM tert-Butyl hydroperoxide (t-BOOH) (**b**) were added 30 min after beginning of NAC or NACET treatment. The percentage refers to 100% viability of untreated cells (mean ± SD, number of replicates = 3). The *p*-values for the comparison of treated vs. untreated cells (point 0) are indicated in black, while *p*-values for comparison between cells treated with equal concentrations of NAC or NACET are indicated in red (** *p* < 0.01; **** *p* < 0.0001; ns, not significant).

NAC has been widely investigated as a potential therapeutic drug for different pathological conditions based on an increase in oxygen radicals. Still, its efficacy is clearly limited by its low cellular and tissue bioavailability [13,14]. To compare the efficacy of NACET versus NAC to protect RPE cells from an oxidative insult, we first treated confluent ARPE-19 cells with increasing concentrations of these drugs, starting with 0.1 mM up to 5 mM for 24 h. Under concomitant oxidative stress induced by 2 mM H_2_O_2_, NAC was found to be protective for RPE cells starting from the concentration of 2 mM, while NACET induced a strong and significant effect already at a concentration of 0.4 mM (Figure 1a). Moreover, NACET treatment also resulted in an enhanced protective effect when RPE cells were stressed by the organic ROS generator peroxide tert-Butyl hydroperoxide (t-BOOH) (Figure 1b).

### 2.2. NACET Increases the GSH Level in RPE Cells

**Figure 2:**
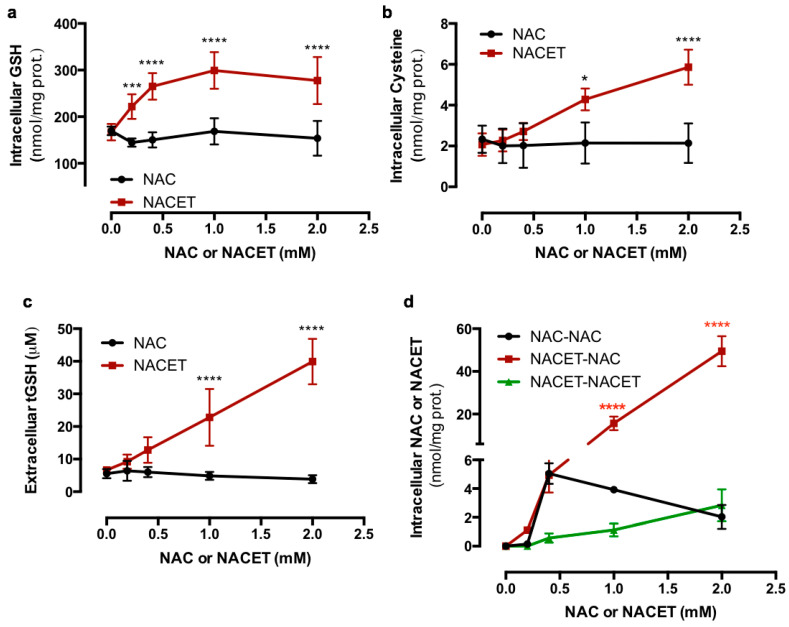
NACET strongly increases the intracellular glutathione (GSH) content in RPE cells. ARPE-19 RPE cells were treated for 16 h with increasing concentrations of NAC or NACET and intracellular concentrations of GSH (**a**), cysteine (**b**), and NAC and NACET (**d**), and the extracellular concentrations of total GSH (tGSH) (**c**) were measured. The intracellular thiol concentrations were normalized to the concentration of total proteins (mean ± SD, number of replicates = 3). The *p*-values for the comparison of the treated vs. untreated cells (point 0) are indicated in black, while the *p*-values for the comparison between the cells treated with equal concentrations of NAC or NACET are indicated in red. * *p* < 0.05; *** *p* < 0.001; **** *p* < 0.0001.

To evaluate the increase in GSH synthesis in RPE cells following exposure to NAC and NACET, we treated ARPE-19 cells for 24 h with increasing drug concentrations and measured the intracellular and secreted GSH and its precursor cysteine. Figure 2 shows that NACET significantly increased both intracellular GSH (Figure 2a) and cysteine (Figure 2b) at 0.2 and 1 mM concentrations, respectively, as well as extracellular total GSH (Figure 2c), while NAC was not able to promote a significant increase in GSH synthesis at any concentration tested. Moreover, the intracellular levels of NAC and NACET were measured, and we observed that NACET, which inside the cell is immediately de-esterified to NAC, was much more effective in rising the NAC concentration than NAC by itself (Figure 2d).

### 2.3. NACET Increases the GSH Level in Rats’ Eyes

**Figure 3:**
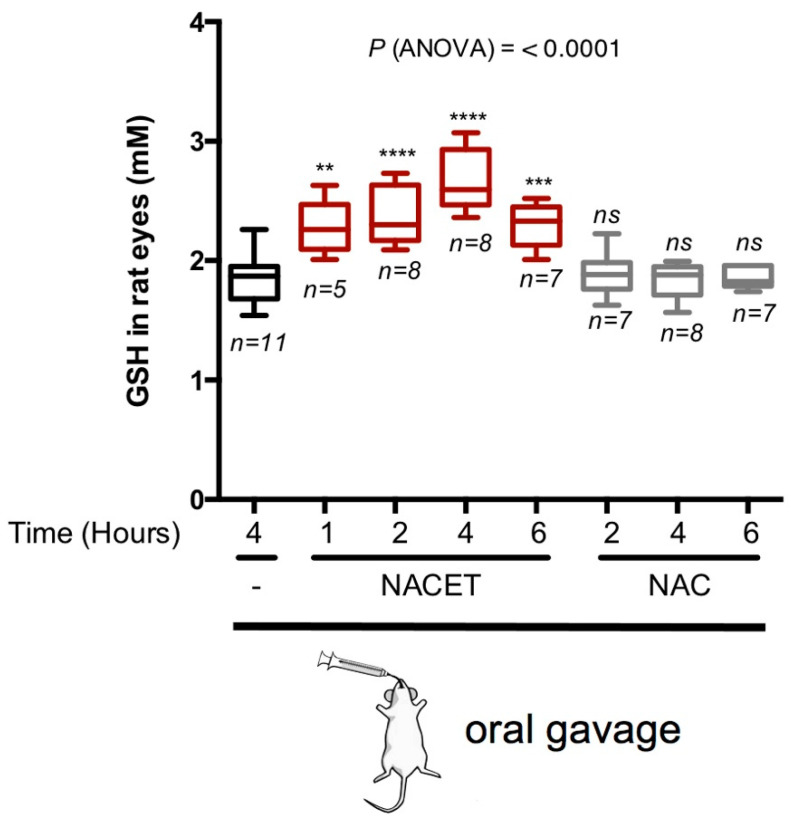
NACET strongly increases the intracellular GSH content in rats’ eyes. The levels of the GSH concentration in the eyes were measured at the indicated time points after oral administration of 50 mg/kg of NAC, NACET, or the vehicle alone. The concentrations were normalized to eye volumes. Data are presented as box and whisker plots displaying the median, lower, and upper quartiles (boxes) and the minimum–maximum (whiskers). Asterisks indicate significant differences (*p*-values) between the data in control animals (gavaged water only; “-”) and each treatment. *n*, number of eyes analyzed; ** *p* < 0.01; *** *p* < 0.001; **** *p* < 0.0001; ns, not significant differences.

The clinical application of many antioxidants is limited due to their poor bioavailability [15,16]. To assess if NACET is able to increase the antioxidant potential in eye tissues in vivo, we orally administrated 50 mg/kg of NAC or NACET to adult rats and evaluated the GSH concentration in the eyes. The GSH level was significantly increased by NACET treatment, with a peak at 4 h following drug administration. In contrast to NACET, NAC did not cause any significant effect on the GSH concentration in the eyes (Figure 3). 

### 2.4. NACET Strongly Increases the Intracellular GSH Pool Available to Counteract Oxidative Stress

**Figure 4:**
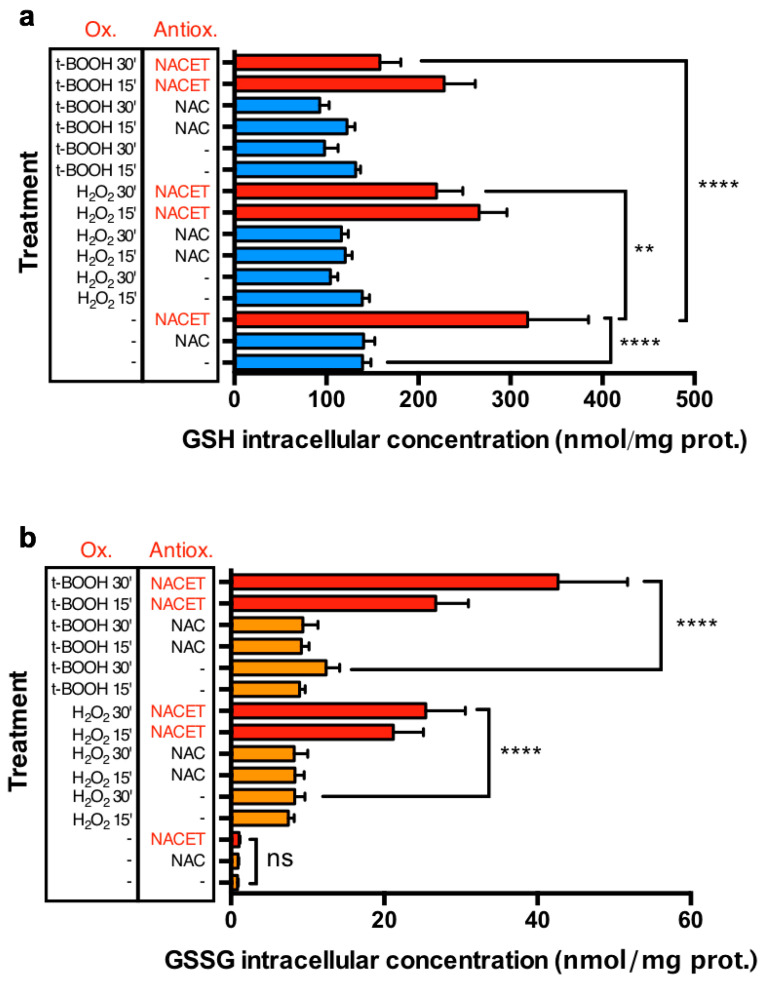
NACET strongly increases the intracellular GSH pool available to counteract oxidative stress. ARPE-19 RPE cells were treated for 16 h with 1 mM antioxidant (Antiox.) and 15 or 30 min before lysis were stressed with 2 mM H_2_O_2_ or 1 mM t-BOOH as oxidants (Ox.). GSH (**a**) and glutathione disulfide (GSSG) (**b**) concentrations were normalized to the concentration of total proteins (mean ± SD, number of replicates = 3). In panel (**a**), GSH concentrations are represented as blue bars for NAC-treated and untreated samples, and as red bars for NACET-treated samples. In panel (**b**), GSSG concentrations are represented as orange bars for NAC-treated and untreated samples, and as red bars for NACET-treated samples. ** *p* < 0.01; **** *p* < 0.0001; ns, not significant differences.

To test if the NACET-induced increase in the GSH concentration is functional for promoting the natural antioxidant defense of cells, we simultaneously measured the levels of intracellular GSH and its oxidized form, glutathione disulfide (GSSG), following NAC or NACET treatment in the presence of H_2_O_2_ or t-BOOH. In ARPE-19 cells, H_2_O_2_ and t-BOOH induced a significant reduction of intracellular GSH and a consequent increase in GSSG levels. In these experimental conditions, only NACET induced a strong and significant increase in the GSH concentration (from 139 ± 9 to 319 ± 66 nmol/mg of proteins) inside the cells (Figure 4a). Upon short H_2_O_2_-induced and t-BOOH-induced oxidative insult, a detectable, but not significative, decrease in GSH was measured in untreated or NAC-treated cells, while a much more important and significant decrease was observed in NACET-treated cells. Conversely, in unstressed cells, the GSSG levels were not influenced by NACET, nor by NAC, but they were strongly and similarly increased after induction of oxidative stress in untreated and NAC-treated cells (e.g., from 0.85 ± 0.11 to 8.33 ± 1.31 nmol/mg of proteins in untreated vs. 30 min H_2_O_2_ stressed cells) (Figure 4b). Interestingly, in NACET-pretreated cells, the increase in the oxidant-induced GSSG levels was much more pronounced, in line with the stronger decrease in GSH concentration, suggesting that NACET promotes an increase in the intracellular GSH pool available to act as a natural antioxidant defense.

### 2.5. NACET Pretreatment Protects RPE Cells from Oxidative Stress

In the experiments described above, NAC and NACET exerted their protective effect both by directly reacting with the oxidative stress generators and by supplying cysteine for the synthesis of glutathione. To compare their reactivity, we incubated NAC or NACET with H_2_O_2_ or t-BOOH and measured the half-life of thiols by reaction with 5,5′-dithiobis (2-nitrobenzoic acid) (DTNB). We found that NACET is much more reactive than NAC, both in the presence of H_2_O_2_ (t_1/2:_ 1.16 ± 0.18 min vs. 8.81 ± 0.45 min) and t-BOOH (t_1/2_: 12.2 ± 0.8 min vs. 88.3 ± 4.51 min) (Table 1).

**Figure 5:**
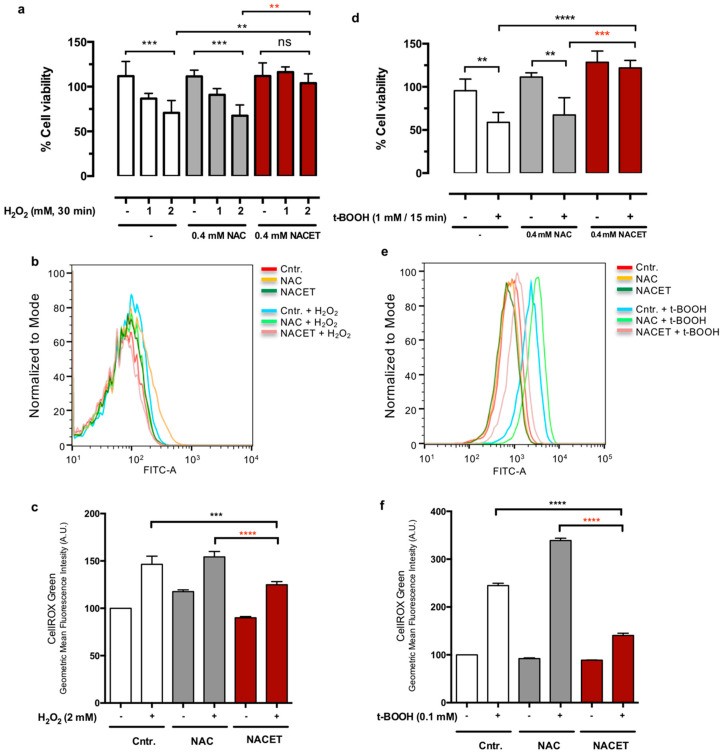
NACET pretreatment predisposes RPE cells to oxidative stress resistance. Cells were incubated for 16 h with NAC or NACET, washed with phosphate-buffered saline solution (PBS) and treated with H_2_O_2_ for 30 min (**a**–**c**) or with t-BOOH for 15 min (**d**) or 1 h (**e,f**) at the indicated concentrations. Viability was measured after 16 h (**a**,**d**) (mean ± SD, number of replicates = 3). The *p*-values for the comparison of the treated vs. untreated cells (point 0) are indicated in black, while the *p*-values for the comparison between the cells treated with equal concentrations of NAC or NACET are indicated in red. Ns, not significant differences. (**b**,**c**,**e**,**f**) The reactive oxygen species (ROS) content was measured by CellROX Green dye staining and FACS analysis immediately after H_2_O_2_ or t-BOOH treatments (mean ± SD, number of replicates = 3). ** *p* < 0.01; *** *p* < 0.001; **** *p* < 0.0001.

To test if NAC and NACET can work as protective agents against oxidative stress acting inside the RPE cells, we pretreated cells with NAC or NACET and removed the compounds before adding the oxidant insult as a short pulse. Figure 5 shows that pretreatment with 0.4 mM NACET, but not an equivalent concentration of NAC, was able to predispose cells to counteract the decrease in RPE cell viability induced by both H_2_O_2_ (Figure 5a) and t-BOOH (Figure 5d). To demonstrate that these effects were due to a reduced ROS content in NACET-pretreated cells, we performed a CellROX Green dye staining and Fluorescence-activated Cell Sorting (FACS) analysis immediately after H_2_O_2_ or t-BOOH treatments and observed a reduction in the amount of ROS generated inside the cells by oxidative stress (Figure 5b,c,e,f). 

## 3. Discussion

Light-induced oxidative stress and the accumulation of free radicals during aging are considered the main triggers of the AMD pathogenesis. GSH is an essential intra- and extracellular protective antioxidant against oxidative stresses, and retinal GSH levels are known to decrease in response to light exposure [17]. Therefore, exogenous thiol replenishment, e.g., through NAC treatment, is under study as a method for reducing the oxidative damage in AMD [12]. Herein, we demonstrated that NACET, a lipophilic cell-permeable cysteine derivative, acts as a protective agent against oxidative damage at a 5–10 times lower concentration than NAC. Moreover, at the concentrations tested, NAC was not able to induce in RPE cells an increase of intra- or extracellular GSH, despite being a cysteine prodrug, and cell pretreatment with NAC did not prevent intracellular ROS formation following oxidative insult. These observations did not seem in agreement with many others in the literature, where NAC has been described as an efficient antioxidant compound. This can be easily explained by the fact that NAC is often used concurrently with pro-oxidant agents and can reduce oxidative stress, most probably by reacting directly with them in the cell culture medium, and even when a pretreatment is declared, in most cases NAC is not washed away before the oxidative insult. NACET can act in the same way and, at least for the oxidants used here, more efficiently by reacting faster, with a t_1/2_ more than seven times lower than that of NAC. Notably, herein, we found that NACET maintained its capability to protect cells against oxidative stress even when pretreatment was followed by extensive washing, suggesting that NACET increases the internal defense mechanisms of RPE cells to cope with oxidative stress. This happened most probably by efficiently increasing a subcellular pool of GSH available for this aim, as demonstrated by the substantially higher consumption of GSH and production of its oxidized form, GSSG, in NACET-pretreated cells subjected to oxidative insult in comparison with untreated or NAC-pretreated cells. The conventional antioxidant supplements that are studied do not effectively prevent and certainly do not stop the progression of AMD [10,18]. These disappointing results are most probably due to the low permeability of the mitochondria, where ROS are mainly produced, and to blood–retinal barrier. To circumvent the difficulty of low mitochondrial permeability, many efforts have been made to develop mitochondrial-targeted antioxidants, which are considered a promising intervention in age-related ocular diseases [19,20]. In this context, NAC ester prodrugs, such as NACET, with their increased lipophilicity can be very interesting drug candidates. It is worth noting that while this manuscript was in preparation, Kularatne and colleagues described how four ester derivatives of NAC, including NACET, were able to protect RPE cells against oxidative damage induced by treatment with hydroquinone (HQ), a component of cigarette smoke [21]. Moreover, following HQ treatment, N-acetylcysteine butyl ester (NACBE) protected RPE cells from cell–cell junction disruption and, in general, ester derivatives of NAC prevented mitochondrial oxidative damage, measured as mitochondrial depolarization, even better than MitoQ, a well-known mitochondrial-targeted antioxidant. Interestingly, NACBE was described as increasing the mitochondrial GSH pool, and this feature is most likely shared by NACET and other NAC ester derivatives, since they have common physical and chemical characteristics. 

Even if treatment of posterior eye diseases causing irreversible blindness, such as AMD, diabetic retinopathy, or retinitis pigmentosa (RP), is urgent, these disorders are characterized by difficulty in their treatment due to the blood–retinal barrier, which strictly regulates drug permeation from the blood to the retina [16]. NAC and many natural antioxidants failed to obtain effective results in vivo due, in part, to their relatively poor bioavailability and pharmacokinetics [15]. In a preliminary phase I clinical trial, NAC modestly but significantly improved the suboptimally functioning macular cones in RP patients [22]. In that trial, to reach this goal, high NAC doses were administered to patients with the registration of gastrointestinal adverse events and a considerable variability in aqueous and plasma NAC, probably only because of its relatively low absorption and bioavailability [13]. Thus, another important observation of our study was the increase in GSH in the eyes of animals that orally received NACET, when NAC failed. Hence, the lipophilicity of NACET, and in general of ester derivatives of NAC, not only has the advantage of promoting their entry into cells and mitochondria, where they produce NAC and cysteine [13,21], but also of remarkably enhancing their tissue absorption and of crossing various blood barriers [13].

**Figure 6:**
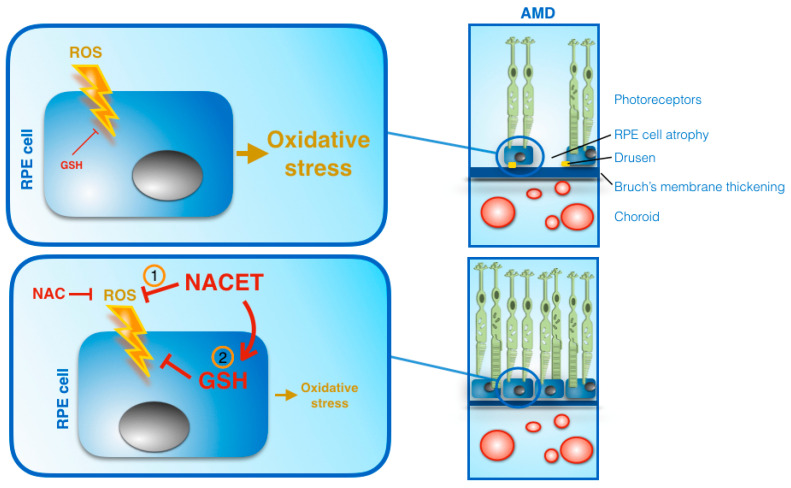
Schematic view of the mechanisms of NAC and NACET on promoting RPE cells’ resistance to oxidative stress. NACET was more effective than NAC because it acted directly by reacting more rapidly with ROS (1) and within the cell by increasing the concentration of available GSH (2). The arrowhead lines represent positive influences, flat-ended lines indicate negative influences. AMD, age-related macular degeneration.

Taken together, all of these observations suggest that NACET, or other ester derivatives of NAC, by directly reacting with ROS and through an increase of GSH in the eye, may represent a pool of promising weapons against oxidative stress that induces AMD or other degenerative photoreceptor diseases (Figure 6). Targeted experiments in animal models of such diseases are still missing and are urgently needed to investigate the impact of these drugs on the prevention and/or suppression of the progression of degenerative retinal diseases.

## 4. Materials and Methods

### 4.1. Cell Cultures and Treatments

Human retinal pigment epithelial cell line ARPE-19 was grown in DMEM/F12 (1:1) containing 10% (*v*/*v*) fetal bovine serum (FBS) and penicillin and streptomycin (all reagents were purchased from Euroclone, Milan, Italy). Cells were free of mycoplasma, confirmed with the MycoAlert mycoplasma detection kit (Lonza, Walkersville, MD, USA). ARPE-19 viability was evaluated as previously described [23]. Briefly, ARPE-19 cells were plated in a 96-well plate (1 × 10^4^ cells per well), grown at confluence and starved in 1% (*v*/*v*) FBS for 48 h. The medium was changed before treatments and cell viability was quantified 16 h later. The final concentrations of H_2_O_2,_ tert-Butyl hydroperoxide (t-BOOH), NAC (Sigma Aldrich, Milan, Italy), and NACET, and the duration of the treatments are indicated in the figure legends. The synthesis, purification and mass spectrometry, ^1^H NMR, infrared spectrometry, and polarimetry characterization of NACET (C_7_H_13_NO_3_S, MW 191.2, mp 44.1–44.5 °C) have previously been reported [24]. Briefly, NACET was prepared under argon atmosphere by N-acetylation of L-cysteine ethyl ester (Merck, Darmstadt, Germany) in dichloromethane with equimolar amounts of acetic anhydride (Merck, Darmstadt, Germany). HPLC analysis with UV (215 nm) absorbance detection of the isolated product revealed a chemical purity of >99% for NACET.

Cell viability was quantified by using the CellTiter 96^®^ AQ_ueous_ one-solution cell proliferation kit (Promega, Fitchburg, WI, USA). Stock solutions (20 mM) of the drugs (NAC and NACET) were prepared the day of the experiment in H_2_O and then diluted to the final concentration in culture medium.

### 4.2. In Vivo Treatments and Animal Manipulation

Male Sprague-Dawley rats (250–300 g) were obtained from Envigo RMS (Udine, Italy). The animals were kept under controlled conditions (22–24 °C, relative humidity 40–50%, under a 12 h light/dark cycle) and fed ad libitum for 2–3 weeks before their use. Rats were dosed by oral gavage with 1 mL of deionized H_2_O (negative controls), NAC solution (50 mg/kg), or NACET solution (50 mg/kg). At the indicated time points, the rats were sacrificed, and their eyes were enucleated, cleaned of extraocular tissue, and weighed. Rapidly, the eyes were homogenized in 500 µL of ice cold 4% (*w*/*v*) trichloroacetic acid (TCA) (Sigma Aldrich, Milan, Italy) and homogenized in ice by means of a tissue grinder. Samples were immediately analyzed and the GSH concentration normalized for eye volumes.

### 4.3. Analysis of Intracellular and Extracellular Low Molecular Mass Thiols (LMM-SH)

In those experiments where the intracellular levels of GSH, NAC and NACET were measured, the culture medium was removed and the cells were washed twice (1 min each) with phosphate-buffered saline solution (PBS), pH 7.4, at 4 °C, lysed by treatment with 0.5 mL of a 4% (*w*/*v*) solution of ice-cold TCA containing 1mM K3EDTA and collected after scraping. Samples were either immediately analyzed or stored at −80 °C until analyses. Measurements were always carried out within 5 days from sample preparation. Thiol analyses were performed by HPLC after labeling of the –SH group with the fluorescent probe monobromobimane (mBrB) (Calbiochem, La Jolla, CA, USA) as previously described [14]. The same HPLC method was also applied to supernatants of homogenized rats’ eyes to measure their LMM-SH content.

Analyses of the total thiols in the culture medium were carried out by the same HPLC method after reduction of disulfides with 2 mM dithiothreitol (DTT).

In those experiments where the intracellular levels of GSH and GSSG were simultaneously measured, a pretreatment with *N*-ethylmaleimide (NEM) was carried out [25]. Briefly, cells were washed twice with 5 mM NEM in PBS and lysed by the addition of 4% (*w*/*v*) TCA containing 1 mM K_3_EDTA. GSH was measured in the supernatant by UV-Vis HPLC and GSSG by fluorometric HPLC after DTT reduction and labeling with mBrB [22]. An Agilent series 1100 HPLC (Agilent Technologies, Milan, Italy) equipped with diode array and a fluorescence detector was used for all determinations. All expression analyses were performed three times independently.

### 4.4. Measurement of NAC and NACET Reactivity

NAC or NACET was reacted with 10 mM (final concentration) H_2_O_2_ or t-BOOH at pH 7.4 at room temperature. At interval times, the concentration of the –SH groups was detected by reaction with 5,5′-dithiobis (2-nitrobenzoic acid) (DTNB) [26]. The half-life was calculated as 0.693/k, where k is the constant for a pseudo-first-order reaction.

### 4.5. Protein Analysis

For the normalization of the GSH concentrations, protein content was measured by the Bradford assay after protein pellet resuspension in 0.1 N NaOH. Bovine serum albumin was used as standard.

### 4.6. Intracellular ROX Analysis Measurement

ROS analysis was assessed in living cells by using the fluorogenic probe CellROX^®^ Green Reagent (#C10444; Thermo Fisher Scientific Inc., Waltham MA, USA) according to the manufacturer’s protocols. Cells were seeded into 12-well plates (1 × 10^5^ cell/well), treated with 0.4 mM NAC, 0.4 mM NACET, or the vehicle for 16 h, washed with PBS three times and treated with 2 mM H_2_O_2_ or 0.1 mM t-BOOH for 30 min before staining. ROS levels were determined using a FACSCanto II flow cytometer (BD Biosciences, San Jose, CA, USA) and the data were analyzed using FlowJo software (V. 10.0.7r2, FlowJo LLC, Ashland, OR, USA). All analyses were performed in triplicate.

### 4.7. Statistical Analysis 

The data analysis was performed using Prism 6 statistical software (GraphPad Software Inc., San Diego, CA, USA). Evaluation of the data was conducted by one-way or two-way ANOVA. Significant differences were estimated using Tukey’s multiple comparison test (* *p* < 0.05; ** *p* < 0.01; *** *p* < 0.001; **** *p* < 0.0001). Student’s *t*-test was used to confirm significant differences between treatments. Two-tailed probabilities of less than 0.05 were considered significant. 

## Figures and Tables

**Table 1:** Oxidation kinetic of NAC and NACET: 1 mM NAC or NACET was reacted with 10 mM H_2_O_2_ or t-BOOH at room temperature for the indicated time points. The concentration of the –SH groups was detected by reaction with 5,5′-dithiobis (2-nitrobenzoic acid) (DTNB). The half-life was calculated as 0.693/k, where k is the constant for a pseudo-first order reaction. The results are presented as mean ± standard deviation of two–three independent experiments.

Time (min)	NAC (µM) +H_2_O_2_	NACET (µM) +H_2_O_2_	NAC (µM) +t-BOOH	NACET (µM) +t-BOOH
0′	890.7 ± 13.3	827.3 ± 28.5	902.7 ± 24.1	865.3 ± 24.2
2′		281.0 ± 21.6		
4′		84.9 ± 8.8		
6′		21.0 ± 4.6		
8′		5.4 ± 3.5		
10′	401.0 ± 3.6		855.2 ± 8.4	507.7 ± 10.1
15′			844.0 ± 11.3	
20′	193.0 ± 15.7		793.0 ± 3.9	265.3 ± 16.3
30′	84.9 ± 9.2		730.7 ± 16,7	170.0 ± 26.2
40′				112.5 ± 11.9
45′	49.2 ± 1.3		642.7 ± 16.2	68.4 ± 1.5
50′	22.4 ± 1.8			58.3 ± 2.7
60′	13.5 ± 2.6		571.0 ± 9.5	26.3 ± 4.5
120′			368.5 ± 14.8	
240′			144.5 ± 10.6	

## Data Availability

The data presented in this study are available in the manuscript itself.

## Abbreviations

AMD	Age-related macular degeneration
RPE	Retinal pigment epithelium
ROS	Reactive oxygen species
GSH	Glutathione
GSSG	Glutathione disulfide
NAC	N-acetyl-L-cysteine
NACET	N-acetyl-L-cysteine ethyl ester
t-BOOH	Tert-Butyl hydroperoxide
TCA	Trichloroacetic acid
